# Secondary Sclerosing Cholangitis in Critically Ill Patients Alters the Gut–Liver Axis: A Case Control Study

**DOI:** 10.3390/nu12092728

**Published:** 2020-09-07

**Authors:** Andreas Blesl, Christoph Jüngst, Frank Lammert, Günter Fauler, Florian Rainer, Bettina Leber, Nicole Feldbacher, Silvia Stromberger, Renate Wildburger, Walter Spindelböck, Peter Fickert, Angela Horvath, Vanessa Stadlbauer

**Affiliations:** 1 Division for Gastroenterology and Hepatology, Department of Internal Medicine, Medical University of Graz, Auenbruggerplatz 15, 8036 Graz, Austria; florian.rainer@medunigraz.at (F.R.); nicole.feldbacher@medunigraz.at (N.F.); walter.spindelboeck@medunigraz.at (W.S.); peter.fickert@medunigraz.at (P.F.); angela.horvath@medunigraz.at (A.H.); vanessa.stadlbauer@medunigraz.at (V.S.); 2 Division for Gastroenterology and Hepatology, Department of Internal Medicine, Medical University of Zürich, 8032 Zürich, Switzerland; christoph.juengst@usz.ch; 3 Department of Medicine II, Saarland University Medical Center, Saarland University, 66421 Homburg, Germany; Frank.Lammert@uniklinikum-saarland.de; 4 Institute for Medical and Chemical Laboratory Diagnosis, Medical University of Graz, 8036 Graz, Austria; guenter.fauler@medunigraz.at; 5 Department of Surgery, Division of Transplantation Surgery, Medical University of Graz, 8036 Graz, Austria; bettina.leber@medunigraz.at; 6 AUVA Rehabilitation Clinic Tobelbad, 8144 Tobelbad, Austria; Silvia.Stromberger@auva.at (S.S.); Renate.Wildburger@auva.at (R.W.)

**Keywords:** SC-CIP, secondary sclerosing cholangitis, critical illness, microbiome, gut permeability, bile acids

## Abstract

Secondary sclerosing cholangitis in critically ill patients (SC-CIP) occurs after long-term intensive care treatment. This study aimed to assess the gut–liver axis in SC-CIP. Stool microbiome composition, gut permeability, bacterial translocation and serum bile acid profiles of 18 SC-CIP patients compared to 11 patients after critical illness without liver disease (CIP controls), 21 patients with cirrhosis and 21 healthy controls were studied. 16S rDNA was isolated from stool and sequenced using the Illumina technique. Diamine oxidase, zonulin, soluble CD14 (sCD14) and lipopolysaccharide binding protein were measured in serum and calprotectin in stool. Serum bile acids were analyzed by high-performance liquid chromatography-mass spectrometry (HPLC-MS). Reduced microbiome alpha diversity and altered beta diversity were seen in SC-CIP, CIP controls and cirrhosis compared to healthy controls. SC-CIP patients showed a shift towards pathogenic taxa and an oralization. SC-CIP, CIP controls and cirrhotic patients presented with impaired gut permeability, and biomarkers of bacterial translocation were increased in SC-CIP and cirrhosis. Total serum bile acids were elevated in SC-CIP and cirrhosis and the bile acid profile was altered in SC-CIP, CIP controls and cirrhosis. In conclusions, observed alterations of the gut–liver axis in SC-CIP cannot solely be attributed to liver disease, but may also be secondary to long-term intensive care treatment.

## 1. Introduction

Secondary sclerosing cholangitis in critically ill patients (SC-CIP) is a rare, often rapidly advancing, cholestatic liver disease observed in patients without known liver disease after long-term treatment in an intensive care unit (ICU). Septic, cardiogenic or hypovolemic shock, mechanical ventilation, polytrauma, complicated surgeries, burns and severe (co-)morbidities such as obesity have been described as potential initiating factors [1,2,3]. Usually, long-term ICU treatment with a mean duration of 30 to 40 days precedes the onset of this disease, but in an individual case, development of SC-CIP after only nine days of ICU stay has been reported [1,4].

The pathogenesis of SC-CIP remains widely enigmatic, and several potentially causative factors are of major interest: (i) ischemic injury of the biliary system, (ii) bile cast formation with protein-rich sludge and (iii) impaired innate immunity leading to recurrent biliary infections with bacteria and fungi [5,6]. The disease may cause destruction of the intra- and extra-hepatic biliary system with evolution of strictures leading to biliary-type liver fibrosis. Some cases may rapidly progress to cirrhosis within months, with the need for liver transplantation as the ultimate treatment option [6]. Generally, prognosis is bad and therefore, transplant-free survival is only around 40 months [7]. Antibiotic treatment of bacterial cholangitis and endoscopic removal of biliary casts can occasionally lead to temporary improvement [6], but currently, liver transplantation is the only healing treatment, which, in selected patients, has excellent outcomes, similar to other indications [8,9].

In the last three years, data about the microbial changes of the gut and of other locations of the human body in critically ill patients has emerged [10,11,12,13]. Data about the long-term effects of an ICU stay on the gut microbiome are, however, scarce [13]. Microbiological bile analysis from patients with SC-CIP and primary sclerosing cholangitis (PSC) showed an altered microbiological profile in these two groups, with predominance of drug-resistant organisms in the bile of SC-CIP [14]. Data on the gut microbiome in SC-CIP is not available to date, but other chronic liver diseases have distinct changes in microbiome composition with potential impact on inflammation (non-alcoholic fatty liver disease (NAFLD), alcoholic liver disease, PSC and cirrhosis) [15,16,17,18]. Altered gut microbiome composition is thought to increase intestinal permeability [19]. It is most commonly seen in cirrhosis but was also described in alcohol-induced injury, NAFLD and hepatits C virus (HCV)-mediated liver injury [20,21,22,23,24]. When gut permeability is increased, bacteria from the intestinal lumen can translocate into extraintestinal locations of the body (lymph nodes, blood) and trigger inflammatory reactions, leading to disease progression [16,25].

Previous studies convincingly showed that bile acids have a pivotal impact on the composition of the microbiome and on gut permeability. In addition, the microbiome influences vice versa bile acid amount and composition [26,27]. Since the severity of liver disease was shown to correlate with impaired bile acid secretion, several factors may overlap which significantly hinder exact discrimination of cause and consequence in critically ill patients with liver disease [27]. Gut permeability is regulated via farnesoid-X-receptor (FXR), being most commonly activated by primary bile acids [28,29]. Furthermore, in PSC patients with concomitant inflammatory bowel disease (IBD), the stool bile acid profile seems to allow the separation of such patients from those with solely IBD [30]. Taken together, these findings and arguments led us to hypothesize that SC-CIP patients show alterations in the gut microbiome composition and the serum bile acid profile and that these are associated with elevated gut permeability and bacterial translocation as a basis of a pathogenic gut–liver axis.

Consequently, our current study aim was to compare the gut microbiome, gut permeability, inflammatory response and serum bile acid profile in detail in patients with SC-CIP to three different control groups. We therefore included patients suffering from SC-CIP, patients after an ICU stay without liver disease and two additional control groups consisting of patients suffering from alcohol-induced cirrhosis and of healthy probands at three different centers.

## 2. Materials and Methods

### 2.1. Patients

Between December 2014 and May 2015, patients with SC-CIP at two study centers in Graz, Austria, and Homburg, Germany, were recruited from the outpatient liver clinics for this study. Exclusion criteria were other potential causes of cholestasis, such as choledocholithiasis, primary sclerosing cholangitis, primary biliary cholangitis, IgG4-associated cholangitis or toxic cholestasis. Diagnosis was made because of typical cholangiographic findings like irregular intrahepatic bile ducts with strictures, prestenotic dilations, bile duct rarefication or biliary casts on endoscopic retrograde cholangiopancreatography (ERCP) or equivalent findings on magnetic resonance cholangiopancreatography (MRCP) [1]. None of the probands underwent biliary surgery or had a history of IBD. After informed consent was obtained, stool and blood samples were collected at a single time point. The study was approved by the research ethics committees at the Medical University of Graz (26-569 ex 13/14) and Ärztekammer des Saarlands (177/15), and the study was registered at clinicaltrials.gov (NCT02545309).

The first control group (CIP controls) includes patients who were also suffering from severe illnesses with the need for intensive care treatment, but who did not develop liver complications. These patients were recruited between July 2018 and January 2019 at the rehabilitation center Tobelbad near Graz (35/2017 AUVA ethics committee). Patients in this cohort had to have at least two days of invasive ventilation and treatment with vasoactive drugs. Exclusion criteria included known liver and gut pathologies, elevated serum alkaline phosphatase levels and laboratory findings suggesting liver damage or cirrhosis. Stool and blood samples were taken at a single time point after informed consent was obtained.

Patients for the second control group (cirrhosis) with alcohol-induced cirrhosis were matched concerning age and sex from an earlier recruited cohort of our study group. These patients were enrolled at the outpatient clinic at the University Hospital of Graz, Austria, for an intervention study between July 2012 and September 2013. The study protocol was authorized by the ethics committee in Graz (23-096 ex 10/11) and registered at clinicaltrials.gov (NCT01607528). Diagnosis of cirrhosis was based on liver histopathological examinations or a mixture of clinical, radiological and/or laboratory features. Alcohol intake within two weeks before inclusion, active infection at screening, gastrointestinal bleeding within two weeks before inclusion, immuno-modulating drugs, hepatic encephalopathy stage two or higher, renal failure (creatinine over 1.7 mg/dL), other severe diseases unassociated to cirrhosis, malignancy or pregnancy were excluded. Samples were taken before any intervention took place. Within this protocol, also healthy controls (healthy) without liver or gut pathologies were recruited at the Medical University of Graz from October to December 2014.

All study procedures were performed according to the declaration of Helsinki after written informed consent was obtained.

### 2.2. Liver Stiffness and Laboratory Assessments

Liver stiffness of SC-CIP patients was evaluated by elastography on a Fibroscan 502 touch (Echosens, Paris, France). Since no cut-offs for staging of liver fibrosis in SC-CIP exist, those for PSC were used. Cirrhosis was defined by values of more than 14.3 kPa. The cut-off for liver fibrosis was 8.8 kPa [31]. Full blood count, liver enzymes, coagulation parameters and c-reactive protein were measured in the routine laboratory.

### 2.3. Microbiome Analyses

Patients received written instructions for stool collection and immediate transport to the study center or stool samples were directly picked up by the study team. Stool samples were frozen at −80 °C upon arrival in the study center. Total DNA was extracted from frozen stool samples using the MagnaPure LC DNA Isolation Kit III (Bacteria, Fungi) (Roche, Mannheim, Germany) according to the manufacturer’s directive including mechanic and enzymatic lysis [32]. Hypervariable regions V1–V2 were amplified in a target-specific PCR using the primers 27F and R357 (27F-AGAGTTTGATCCTGGCTCAG; R357-CTGCTGCCTYCCGTA) and sequenced with the Illumina MiSeq technique (Illumina, Eindhoven, The Netherlands) [32]. Resulting FASTQ files were taken for data analysis. Sequencing was done with the help of the Core Facility for Molecular Biology at the Center for Medical Research in Graz.

### 2.4. Gut Permeability Markers

Enzyme-linked immunosorbent assays (ELISA) were used to measure calprotectin, zonulin and serum diaminoxidase (DAO) (Immundiagnostic AG, Bensheim, Germany). Lipopolysaccharide-binding protein (LPB) and soluble CD14 (sCD14) levels were identified via a ready-to-use solid-phase sandwich ELISA (Hycult biotechnology, Uden, The Netherlands).

### 2.5. Serum Bile Acids

Bile acid analysis was performed as previously described by Amplatz et al. [33] from deproteinized serum using a high-performance liquid chromatography HPLC equipped with a Nucleoshell C18 reversed phase column (Macherey-Nagel, Düren, Germany) followed by identification by a Q Exactive hybrid quadrupole-orbitrap mass spectrometer (Thermo Fisher Scientific, Waltham, MA, USA).

### 2.6. Statistical Analyses

The R package “dada2” was used to process the sequencing data [34,35]. Raw forward and reverse sequences were trimmed to a length of 250 and 200 bases respectively, and low-quality reads were removed from analysis (maximum expected errors = 2). Dada2 core sample inference algorithm was used to infer true sequence variants for each sample individually. Paired reads were then merged, and chimeras were removed. Low abundant features (i.e., less than ten copies found and present in less than three samples) were removed. Taxonomy was assigned using a Naïve Bayes classifier and SILVA v132 as a reference database. Operational taxonomic units (OTUs) were blasted using the NCBI database [36]. Assigned names are based on best match with at least 97% similarity. Species level identification was done using the addSpecies () function, which assigns species names in case of exact matching. Lower accordance was counted as not further definable. After pre-processing, the average count per sample was 113,031 (standard deviation: 56,149; range: 19,479 to 235,167). In total, 7,912,177 sequencing reads with a fraction of non-zero values of 0.113 were obtained. For normalization, Hellinger transformation was applied. Chloroplast and cyanobacteria filtering contaminants were removed. Alpha diversity analysis was done in Calypso 8.84 (http://cgenome.net/calypso/) using Chao1, Shannon Index and Richness on a rarefied feature table (sequencing depth 19,479). To assess differences in alpha diversity between groups, analysis of variance (ANOVA) with Bonferroni correction was used. Beta diversity and influencing factors of microbiome composition were examined with Redundancy Analysis (RDA) based on Bray–Curtis dissimilarity. Confounders with a *p* < 0.1 were considered for a multivariate model using RDA+ in Calypso. Multicollinearity was defined as a variance inflation factor of two or higher. To avoid the potential bias introduced by collinear explanatory variables, parallel alternative models were constructed. Differentially abundant taxa were identified with Analysis of Composition of Microbiomes (ANCOM), a test which considers compositional limitations and reduces false discovery rates, but maintains high statistical power, specifically developed for microbiome analysis. This method uses multiple taxon-to-taxon comparisons and concludes varied abundance based on the number of significant group comparisons relative to other taxa [37]. To select taxa associated with the four different groups, LDA-effect size (LEfSe) was used. Network analysis was based on Spearman’s rho associations between groups and statin intake vs. no statin intake by changing the pairwise correlations into dissimilarities to draw nodes in a two-dimensional principal coordinates analysis (PCoA) plot. Bile acid multivariate analyses were done in PAST (Version 3.26) using non-metric multidimensional scaling (NMMDS) with Bray–Curtis dissimilarity. All other statistical analyses were done in SPSS Version 26 using the Kolmogorov–Smirnov test to check for normal distribution. Student’s t-test and Man–Whitney U test were further used to test for significance comparing two groups. The non-parametric Kruskal–Wallis one-way analysis of variance was used to compare the four groups to each other. Bonferroni correction was used to correct for multiple comparisons. Correlations were calculated using the Pearson correlation coefficient. All tests were performed on a 5% significance level.

## 3. Results

### 3.1. Patient Characteristics

Eighteen patients with SC-CIP, 11 patients after treatment at an ICU without liver pathologies, 21 patients with alcohol-induced cirrhosis and 21 healthy controls were included in the analysis (SC-CIP: mean age 59 ± 13 years, 13 men, mean body mass index (BMI): 27.2 ± 5.3 kg/m^2^; CIP controls: mean age 54 ± 15 years, 8 men, BMI: 25.5 ± 3.0 kg/m^2^; cirrhosis: mean age 58 ± 9 years, 16 men, BMI 27.3 ± 5.0 kg/m^2^; healthy: mean age 58 ± 7 years, 9 men, mean BMI: 25.3 ± 3.1 kg/m^2^). Age, sex and BMI did not differ between the four groups. Table 1 summarizes the patient characteristics.

In SC-CIP, initial diagnosis was made between 2006 and 2013. Patients needed ICU treatment for various reasons including polytrauma, burns and serious internal medicine emergencies, such as myocardial infarction or acute respiratory distress syndrome. Seven patients presented with liver cirrhosis at inclusion (mean liver stiffness 37.6 ± 19.4 kiloPascal (kPa); mean Model of End Stage Liver Disease (MELD) score: 13 ± 5). Liver fibrosis (mean liver stiffness 9.6 ± 0.5 kPa) was present in four patients. All patients had normal liver function prior to the need for ICU treatment, with one patient reporting a history of fully recovered drug-induced liver injury due to tuberculostatic agents in the past. Mean duration of ICU treatment was 42 ± 20 days, mean ventilation time was 28 ± 18 days and mean length of catecholamine treatment was 12 ± 8 days. Five patients needed hemodialysis during their ICU stay. The interval between ICU admission and the diagnosis of SC-CIP was 311 ± 442 days (range 23–1523 days). Details are given in Table 2. None of the included patients died within the follow-up period of 457 ± 394 days. One patient underwent liver transplantation. One patient was still on hemodialysis at inclusion because of chronic kidney insufficiency. Study inclusion and sample acquisition was done 41 ± 20 months after the ICU treatment. At time of inclusion, 12 out of 18 patients in the SC-CIP cohort were treated with ursodeoxycholic acid.

CIP controls were treated at an intensive care unit and did not develop SC-CIP. Reasons for critical illness included mainly traumas with injuries of the spine. Mean time of ICU treatment was 19 ± 12 days and mean ventilation time was 14 ± 9 days. Both ICU treatment duration and ventilation time were shorter compared to SC-CIP. All patients received catecholamine treatment, but length of vasoactive treatment was not possible to be assessed exactly in retrospect. Only one patient needed hemodialysis during the ICU stay. The interval between ICU admission and inclusion in the study was 373 ± 313 days (range 95–907 days). Patients in the cirrhosis group were all suffering from alcohol-associated liver disease with Child-Pugh class A (5 or 6 points) and a mean MELD score of 11.0 ± 2.6. None of them received antibiotics. Healthy controls were free of gut or liver pathologies, but also had no relevant other known illnesses like heart or pulmonary diseases.

### 3.2. Microbiome Analysis

#### 3.2.1. Reduced Alpha Diversity in Liver Disease and CIP Controls

**Figure 1:**
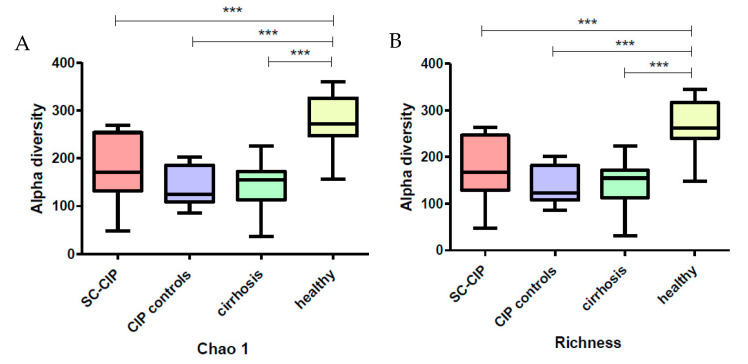
Boxplots showing levels of mean alpha diversity of Secondary Sclerosing Cholangitis in Critically Ill Patients (SC-CIP) (red), patients after critical illness without liver disease (CIP controls) (violet), cirrhosis (green) and healthy (yellow) groups using Chao 1 (**A**) and Richness Index (**B**). Significances are indicated by stars. *** *p* ≤ 0.001. For statistical analysis, analysis of variance (ANOVA) with Bonferroni correction was used.

Alpha diversity using the Chao 1 Index and Richness Index was significantly reduced in SC-CIP, CIP controls and cirrhosis patients compared to healthy controls (*p* < 0.001 for all three groups). No significant difference could be found between SC-CIP, CIP controls and cirrhosis. The Shannon Index only showed reduced diversity in CIP controls and cirrhosis (*p* < 0.05) (Figure 1).

#### 3.2.2. Group Assignment, Statin Use, Cirrhosis, Platelet Function Inhibitor Intake, BMI and Calprotectin as Possible Independent Predictors for Microbiome Composition

**Figure 2:**
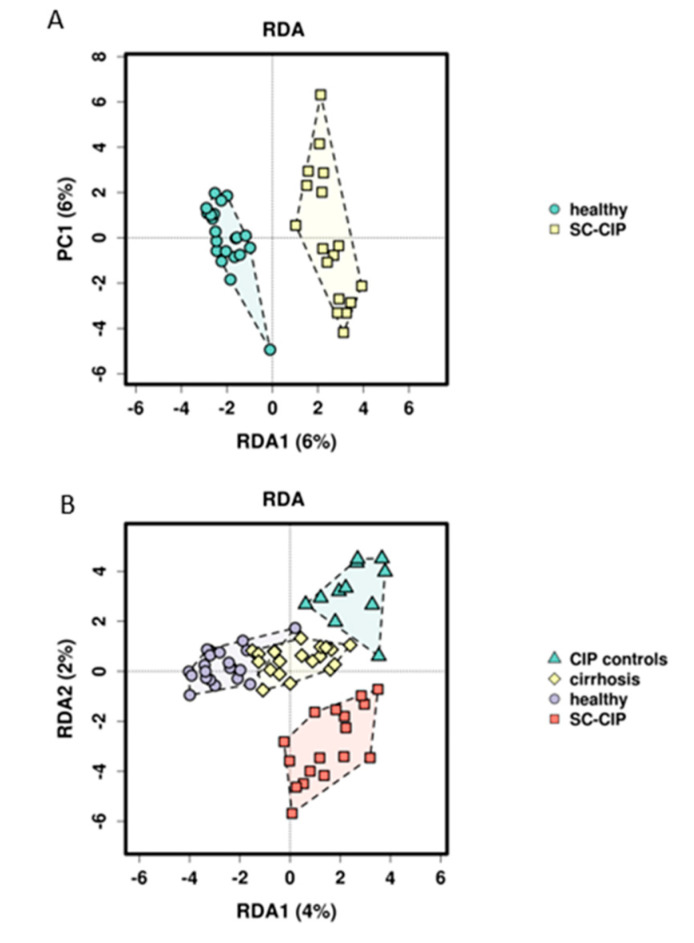
Redundancy analysis (RDA analysis) for beta diversity based on BrayCurtis dissimilarity on the genus level. Each point on the diagram presents one study proband. Each group is drawn in a unique color. (**A**) SC-CIP (yellow) compared to healthy (green) (*p* = 0.001), and (**B**) SC-CIP (red), CIP controls (green), cirrhosis (yellow) and healthy (violet) compared to each other (*p* = 0.001). Findings indicated that with RDA analysis, all four groups showed differences in beta diversity. PC = principal component.

**Figure 3:**
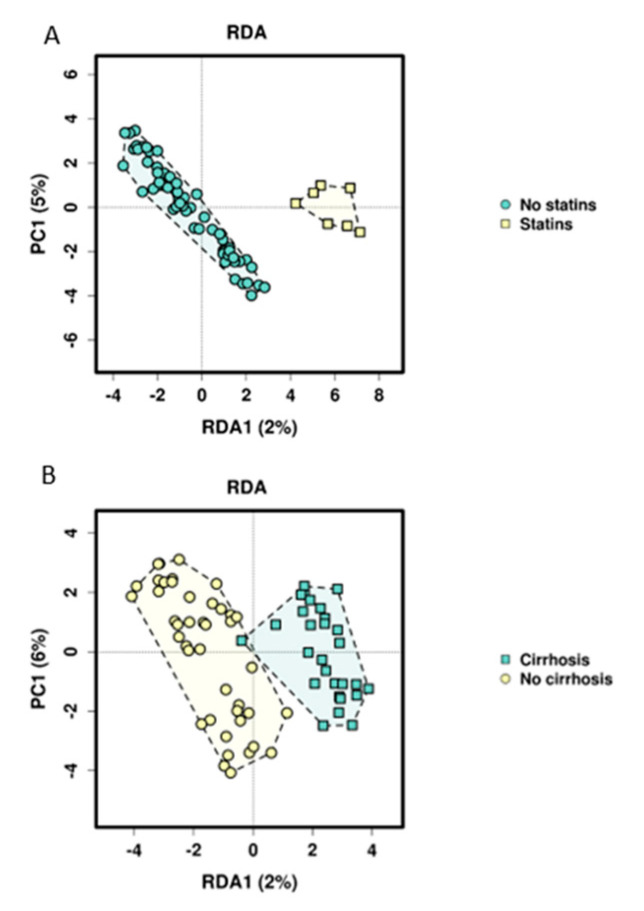
RDA plots for statin intake and cirrhosis. Each point on the diagram presents one study proband. (**A**) People not taking statins are shown in green, people taking statins are shown in orange (*p* = 0.004). (**B**) Patients with cirrhosis are drawn green, controls not suffering from cirrhosis in yellow (*p* = 0.004). In both graphs, clustering of belonging groups can be observed, indicating that cirrhosis and statin intake are possible explanatory variables for microbiome composition.

RDA analysis showed significant differences in beta diversity for all comparisons on the genus level (*p* < 0.001) (Figure 2). To assess influencing factors of microbiome structure on OTU level in addition to group membership, the impact of sex, age, BMI, cirrhosis, c-reactive protein (CRP), creatinine, INR, thrombocytes, bilirubin, markers of gut permeability, intestinal inflammation, bacterial translocation, bile acid composition and intake of the most common drugs (proton pump inhibitors (PPI), antihypertensives, non steroidal anti inflammatory drugs (NSAIDS), statins, beta blockers, thyroid medication and platelet function inhibitors) was assessed. Univariate RDA analysis revealed that cirrhosis, PPI intake, beta blockers, statins, platelet function inhibitors, BMI, CRP, INR, calprotectin, zonulin, DAO, lipopolysaccharide-binding protein (LBP) and sCD14 were potential explanatory variables for microbiome composition with a *p*-value < 0.1. When checking for collinearity with group assignment, cirrhosis and sCD14 showed a Variance Inflation Factor (VIF) greater than 2 as well as low tolerance levels (VIF: cirrhosis, 3.08; sCD14, 2.56). As a consequence, two alternative models were built, one excluding cirrhosis and sCD14 from the list of potential explanatory variables, and one model including cirrhosis and sCD14 but excluding group assignment instead. Multicollinearity was excluded for cirrhosis. The first model (Model 1) identified group assignment (*p* = 0.001) and statin use (*p* = 0.006) as independent predictors of microbiome composition. The second model (Model 2) identified cirrhosis (*p* = 0.003), statins (*p* = 0.001), platelet function inhibitor intake (*p* = 0.001), the BMI (*p* = 0.001) and calprotectin (*p* = 0.022) as independent predictors (Figure 3, Table 3).

**Figure 4:**
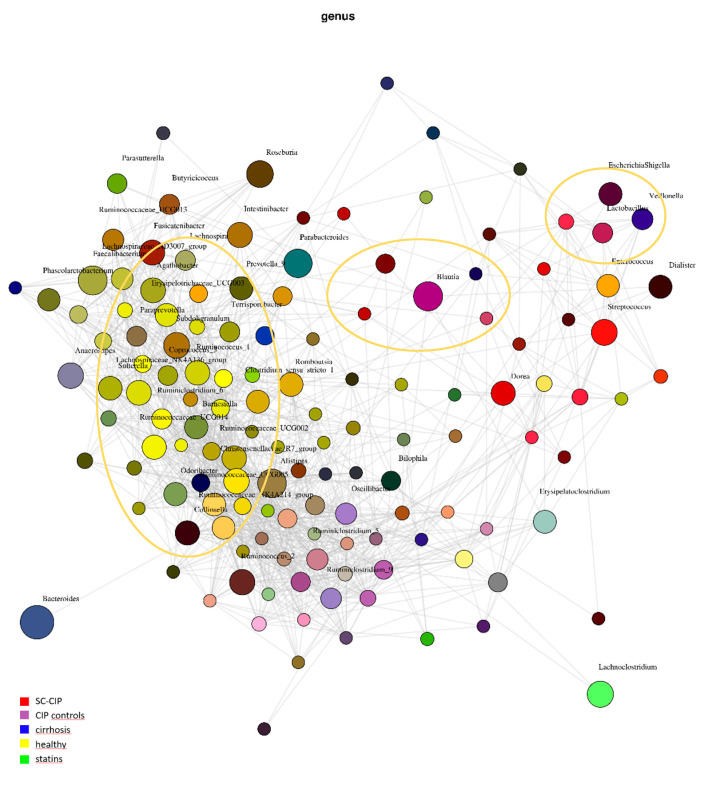
Principal coordinates analysis (PCoA) Plot for Network Analysis for beta diversity using Spearman’s rho correlation. Taxa are drawn as dots, taxa abundance as dot size and edges show positive and negative associations. Green: statin intake, red: SC-CIP, purple: CIP controls, blue: cirrhosis, yellow: healthy. With overlap of colors, between groups similarities of microbiome composition are indicated. The described overlaps of the genera *Blautia* and *Lactobacillus* are marked with purple circles, the close relation of the group with statin intake and healthy is marked with a yellow circle.

To visualize the association of these significant influencing factors, we performed a network analysis including statin intake and group assignment (Figure 4). Similarities of SC-CIP and cirrhosis in the genera *Lactobacillus* and *Blautia* could be visualized by overlap of colors, and patients with statin intake showed close relation to healthy controls.

Further on, SC-CIP and CIP control cohorts were compared to each other to determine factors that favor the development of SC-CIP in these two critically ill patient groups. Variables included in the analysis were duration of ventilation time, length of ICU stay, need for dialysis and catecholamine treatment, as well as all other factors mentioned above. Most likely due the small sample size in both cohorts, only sCD14, bilirubin and the BMI remained significant explanatory variables on univariate RDA analysis. These factors had a Variance Inflation Factor (VIF) smaller than 2 and high tolerance levels, but none of the three remained a significant explanatory variable in the multivariate model (Table 4).

#### 3.2.3. Profound Alterations of the Microbiome in SC-CIP, CIP Controls and Cirrhosis on All Taxonomic Levels

Analysis of composition of microbiomes (ANCOM), as a specific statistical method to analyze microbiome data, was used to assess taxonomic differences from species to phylum levels between all 4 groups. To start, healthy controls were compared with the three disease groups, SC-CIP, CIP controls and cirrhosis. In SC-CIP, on the species level, the inhabitants of the oral cavity *Streptococcus parasanguinis* and *Rothia dentocariosa* as well as the potential pathogen *Enterococcus faecium* and *Streptococcus thermophilus,* which serves as probiotic strain, and *Sellimonas intestinalis*, were elevated compared to healthy controls. The butyrate producing species *Anaerostipes hadrus* showed higher abundance in healthy controls. In CIP controls, the seldom pathogenic *Erysipelatoclostridium ramosum* showed higher abundance compared to healthy controls, whereas *Faecalibacterium prausnitzii*, a potentially beneficial species, showed lower abundance. In cirrhosis, a potential butyrate producing undefined *Coprococcus* and a *Romboutsia* species had lower levels compared to the healthy cohort. Furthermore, multiple differences on genus, family, order, class and phylum levels became evident and are shown in Table 5, Table 6 and Table 7.

In a second step, differences between the three disease groups were evaluated using ANCOM. *Prevotella melaninogenica,* a species inhabiting the oral cavity and causing opportunistic infections, and a not closer defined *Neisseria* species, showed lower abundance in SC-CIP compared to CIP controls. Species of *Neisseria* can cause well-known sexually transmitted diseases and meningitis. *Anaerostipes hadrus* had higher abundance in cirrhosis compared to SC-CIP. Further taxonomic differences on genus, family, order, class and phylum levels are shown in Table 8, Table 9 and Table 10.

#### 3.2.4. Association of the genera *Enterococcus, Streptococcus, Romboutsia, Lactobacillus* and *Butyricicoccus* with SC-CIP

**Figure 5:**
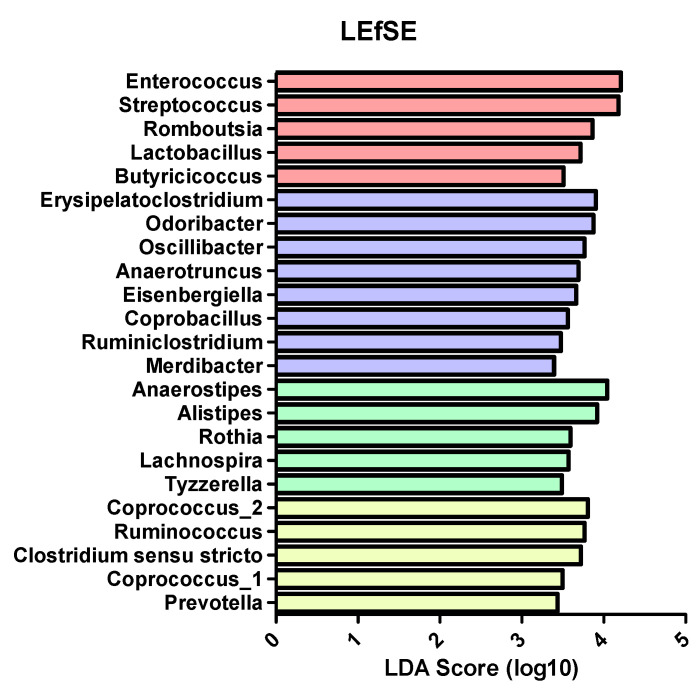
Linear discriminant analysis-effect size (LEfse) results of SC-CIP (red), CIP controls (violet), cirrhosis (green) and healthy (Yellow) groups. The presented genera are associated with microbiome composition of the respective group. The length of the bar shows the logarithmic linear discriminant analysis (LDA) score.

To further investigate microbiome composition in the four distinct groups, we applied supervised machine learning algorithms as a feature selection method on the genus level (LefSE). The oral commensal bacteria *Streptococcus* and *Lactobacillus* as well as *Enterococcus* and *Romboutsia* and the butyrate producing *Butyricicoccus* were found to be associated with SC-CIP. The oral inhabitant *Rothia*, the short-chain fatty acid producing *Anaerostipes* and *Lachnospira*, the genus *Alistipes* which has been related to the development of colorectal cancer and *Tyzzerella,* were found to be associated with cirrhosis. Furthermore, the beneficial genus *Odoribacter*, the cellulosome producing bacterium *Ruminiclostridium*, the important genus for metabolic homeostasis *Merdibacter* as well as *Erysipelatoclostridium, Oscillibacter, Anaerotruncus, Eisenbergiella* and *Coprobacillus* were associated with CIP controls. The butyrate producing *Coprococcus*, *Clostridium senso stricto*, the highly abundant genus *Ruminococcus* and *Prevotella,* which may play a role in the development of abscesses and lives mostly in the oral cavity, were found to be associated with the microbiome of healthy controls (Figure 5).

### 3.3. Altered Gut Permeability and Increased Bacterial Translocation and Inflammation in SC-CIP: Correlation with Liver Damage, Liver Stiffness and Nutrition Status

**Figure 6:**
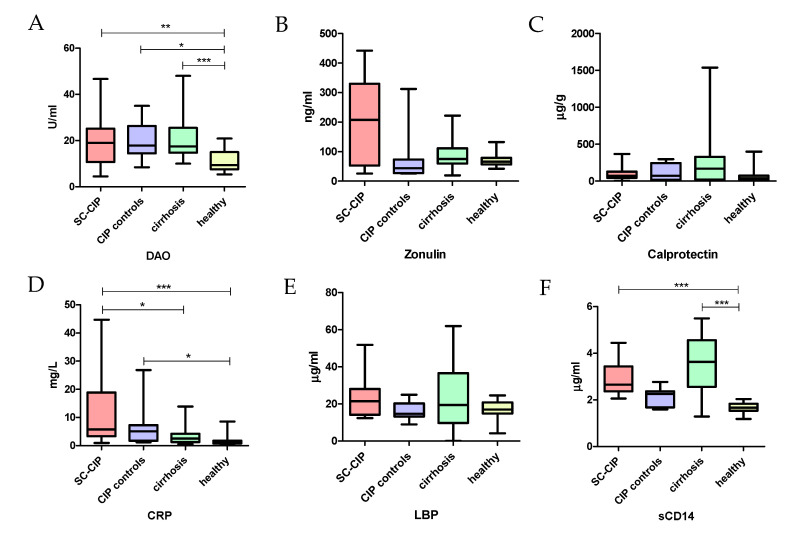
Boxplots for markers of gut permeability, bacterial translocation and intestinal and systemic inflammation. (**A**) diaminoxidase (**B**) zonulin (**C**) calprotectin (**D**) c-reactive protein (**E**) lipopolysaccharide-binding protein (**F**) soluble cluster of differentiation 14. SC-CIP (red), CIP controls (violet), cirrhosis (green), healthy (yellow). Significances are indicated with bars and stars within the diagrams. * *p* ≤ 0.05; ** *p* ≤ 0.01; *** *p* ≤ 0.001. For statistical analysis, analysis of variance (ANOVA) with Bonferroni correction was used; DAO = diaminoxidase; CRP = c-reactive protein; LBP = lipopolysaccharide-binding protein; sCD14 = soluble cluster of differentiation 14.

Serum levels of diaminoxidase (DAO) as a marker of gut permeability were significantly increased in all three disease groups compared to healthy controls (SC-CIP–healthy: *p* = 0.005; CIP controls–healthy: *p* = 0.013, cirrhosis–healthy: *p* = 0.001). Zonulin and calprotectin in stool were not distinct between groups, but CRP was significantly higher in SC-CIP and CIP controls compared to healthy and in SC-CIP patients compared to cirrhosis (SC-CIP–healthy: *p* < 0.001; CIP controls–healthy: *p* = 0.016; Cirrhosis–SC-CIP: *p* = 0.04). LBP as a marker for bacterial translocation was not different in the four groups, but soluble CD14 (sCD14) was significantly elevated in SC-CIP and cirrhosis compared to healthy controls (*p* < 0.001) and in cirrhosis compared to CIP controls (*p* = 0.01) (Figure 6). Significant positive correlation of markers for gut permeability, bacterial translocation and inflammation with liver damage, liver stiffness and nutrition status could be demonstrated (Table 11).

### 3.4. Changed Bile Acid Profiles in SC-CIP, CIP Controls and Cirrhosis: Correlations of Bile Acid Levels with Markers for Bacterial Translocation, Gut Permeability and Liver Damage

**Figure 7:**
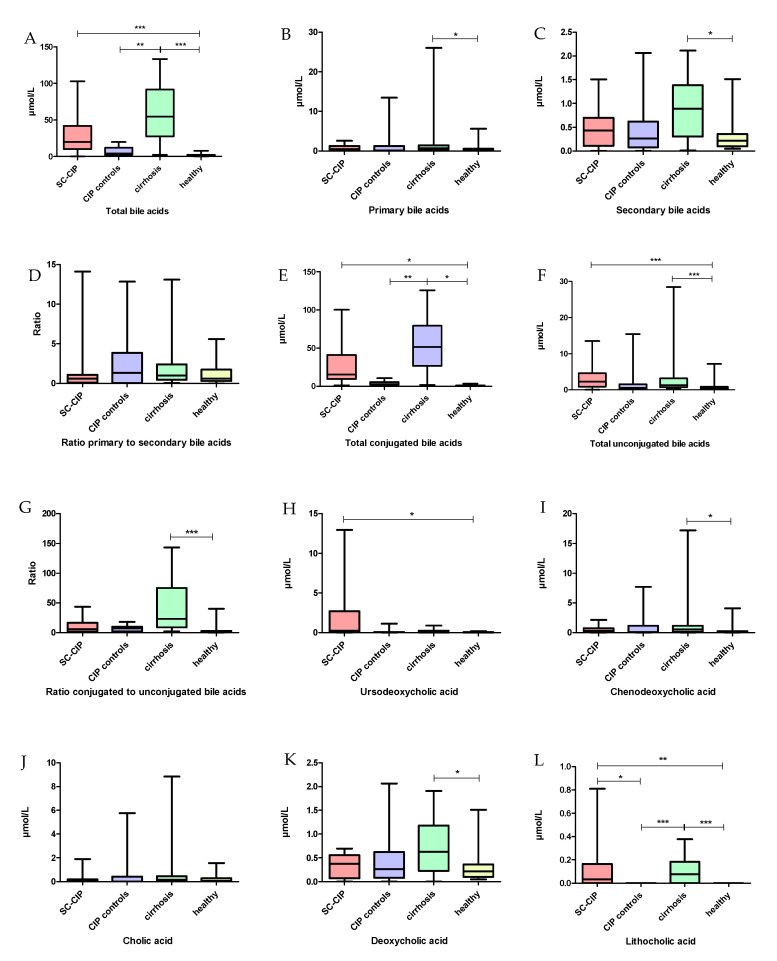
Boxplots for comparison of bile acid levels. (**A**) total bile acids (**B**) primary bile acids (**C**) secondary bile acids (**D**) ratio primary to secondary bile acids (**E**) total conjugated bile acids (**F**) total unconjugated bile acids (**G**) ratio conjugated to unconjugated bile acids (**H**) ursodexocholic acid (**I**) chenodeoxycholic acid (**J**) cholic acid (**K**) desoxycholic acid (**L**) lithocholic acid.SC-CIP (red), CIP controls (violet), cirrhosis (green), healthy (yellow). Significances are indicated with bars and stars within the diagrams. * *p* ≤ 0.05; ** *p* ≤ 0.01; *** *p* ≤ 0.001. For statistical analysis, analysis of variance (ANOVA) with Bonferroni correction was used.

**Figure 8:**
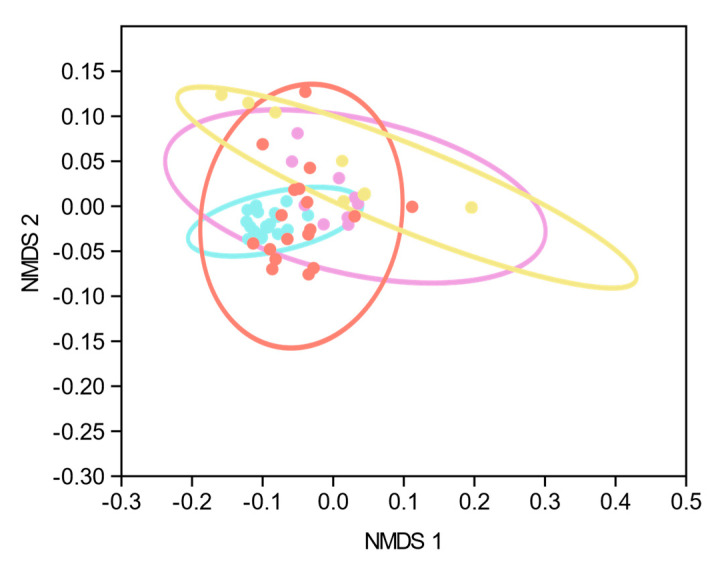
Principal coordinates analysis (PCoA) plot for serum bile acid composition using non-metric multidimensional scaling (Bray–Curtis) for analysis. Orange: SC-CIP, violet: CIP controls, turquoise: cirrhosis, yellow: healthy. Each point on the diagram presents one study proband. Significantly different bile acid profiles were found in healthy compared to SC-CIP, CIP control and cirrhosis groups and between SC-CIP and cirrhosis groups. *R* = 0.26, *p* < 0.001; SC-CIP–healthy: *p* < 0.001; cirrhosis–healthy: *p* < 0.001; CIP controls–healthy: *p* = 0.006. NMDS = Non-metric multidimensional scaling.

The total serum concentration of bile acids was significantly elevated in SC-CIP and cirrhosis compared to healthy controls (*p* < 0.001) and levels were higher in cirrhosis compared to CIP controls (*p* = 0.007). Twelve out of eighteen patients in the SC-CIP group were treated with ursodeoxycholic acid (UDCA) and therefore, UDCA was significantly increased in the SC-CIP cohort compared to healthy controls (*p* = 0.02), but not compared to the other groups, so UDCA was not excluded in the further analysis. Primary and secondary bile acids were elevated in cirrhosis compared to healthy controls (*p* = 0.05; *p* = 0.01) and total conjugated and unconjugated bile acids in SC-CIP and cirrhosis compared to healthy controls (total conjugated both: *p* < 0.001, total unconjugated both: *p* = 0.02). Total conjugated bile acids were higher in cirrhosis compared to CIP controls (*p* = 0.007). On individual bile acid levels, the primary bile acid, cholic acid, was unchanged between the groups, whereas chenodeoxycholic acid was higher in cirrhosis compared to healthy controls (*p* = 0.02). Concerning the secondary bile acids, desoxycholic acid was elevated in cirrhosis compared to healthy controls (*p* = 0.05). Lithocholic acid showed high levels in cirrhosis and SC-CIP and levels close to zero in CIP controls and healthy controls (Figure 7). With multivariate non-metric multidimensional scaling (NMMDS) analysis, significantly altered bile acid profiles were found in healthy compared to SC-CIP, CIP control and cirrhosis groups. The analysis was done with and without inclusion of UDCA (R = 0.26, *p* < 0.001; SC-CIP–healthy: *p* < 0.001; cirrhosis–healthy: *p* < 0.001; CIP controls–healthy: *p* = 0.006). Differences between SC-CIP and cirrhosis were also significant, independent of UDCA inclusion (*p* = 0.03) (Figure 8). Several positive correlations of bile acids with markers for bacterial translocation, gut permeability and liver damage could be found (Table 12).

## 4. Discussion

Since SC-CIP is a relatively young disease entity and its occurrence is, despite growing intensive care treatment possibilities, still rare, sufficient evidence on the pathophysiology of this disease is lacking. Meanwhile, it is common knowledge that other liver diseases show alterations of the gut microbiome, gut permeability and bile acid composition [38]. Still, the connection of these deviations with the underlying liver disease remains uncertain. Are these changes the drivers for the development of the disease or the consequence of the disease itself? To address these questions in SC-CIP, we conducted the present clinical study comparing SC-CIP patients with three different control groups. Applying current state-of-the-art methods, we could demonstrate for the first time that patients with SC-CIP have distinct changes in the stool microbiome composition, disturbed gut permeability, increased bacterial translocation and an altered serum bile acid profile compared to healthy controls. Patients treated in an ICU, who were at risk for SC-CIP, but who did not develop the disease (CIP controls), also show changes of the gut microbiome composition, increased gut permeability, as well as a distinct serum bile acid profile relative to healthy controls, which may be interpreted as long-term effects after ICU stay.

Alpha diversity was significantly reduced in SC-CIP and cirrhosis, a finding which was also observed in other liver diseases, including cirrhosis cohorts [39,40], as well as in obesity, inflammatory bowel disease, antibiotic treatment and ICU patients [41,42,43,44]. Interestingly, CIP controls also showed reduced alpha diversity, highlighting a potential lasting effect of the ICU stay itself on microbial diversity even more than one year after the intensive care treatment. In contrast, data from Aardema et al. suggest a faster recovery of the microbiome in their cohort of ICU patients after cardiac surgery, but the mean ICU stay of these patients was only one day [13].

Using RDA analysis, differential clustering of SC-CIP and CIP controls compared to healthy controls could be observed. Comparable changes in beta diversity have been shown as early as 48 h after admission in ICU patients suffering from critical illnesses of different origins [45] or in patients with early sepsis without organ failure [46]. However, it is currently unclear how long such changes persist. Our data suggests that in SC-CIP, dysbiosis persists, since the mean time span between ICU admission and study inclusion was more than three years. The distinctly different microbiome composition of CIP control compared to healthy controls indicates that these changes persist at least partly from the critical illness and the ICU stay rather than being solely driven by the liver disease itself, and may also be influenced by co-factors like diet and drug intake.

Searching for influencing factors for microbiome composition other than just group assignment, we found that statins significantly influence the microbiome in our study cohort in both applied multivariate models. This influence was also recently shown in a mouse model, indicating changes in the gut microbiome by statins and as a consequence, improving metabolic disorders such as hyperglycemia [47]. Cirrhosis, platelet functions inhibitor intake, the BMI and calprotectin could be identified as other potential microbiome-modulating factors. The link of obesity, nutritional status, diet and microbiome composition has been described and discussed [48,49,50], and the influence of cirrhosis on the microbiome as well as the role of microbiome alterations and signatures as biomarkers for diagnosis and disease progression of cirrhosis have been shown [51]. More recent studies focused on the role of dysbiosis in the emergence of cirrhosis to hepatocellular carcinoma and its potential role as a biomarker for early carcinoma detection [52,53,54,55,56]. The fact that NSAIDs could not be associated with microbiome composition in our study, contrary to previous literature [57], might be caused by the small sample size and the low number of patients taking NSAIDs. Characteristics of the ICU stay could not explain differences in microbiome composition. This may also be due to the relatively small sample size and the long timespan between sample acquisition and ICU treatment.

As expected from the literature [40,58], differences on all taxonomic levels could be observed in SC-CIP and cirrhosis compared to healthy controls, even though the patients in our cohort were stable outpatients with a rather mild course of disease. Only 7 patients were suffering from cirrhosis at the time of the study and none of them had decompensated cirrhosis. Furthermore, the magnitude of differentially abundant species in CIP controls compared to healthy controls was striking, because these patients were included into the study on average more than 1 year after intensive care treatment and did not suffer from liver disease. The dominant phylum observed in all four groups were the phyla Firmicutes and Bacteriodetes, followed by Proteobacteria and Actinobacteria. SC-CIP patients had an increased Firmicutes/Bacteriodetes ratio compared to all three other groups. This increased ratio has recently been associated with obesity, irritable bowel syndrome, leaky gut, NAFLD, cirrhosis and ICU mortality, although it was found not to be a good biomarker for critically ill patients because the Firmicutes/Bacteriodetes ratio may be normal when both phyla are equally affected [38,40,59,60].

In more detail, there seems to be a shift towards pathogenic taxa in SC-CIP compared to healthy controls, highlighted by a high abundance of *Streptococcus* and *Enterococcus* in SC-CIP. High abundances of these genera have previously been observed in cirrhosis, acute on chronic liver failure and NAFLD, and is discussed to influence the clinical phenotype and prognosis [18,38]. Freedberg et al. [61] showed, in their work on the influences of PPI on the microbiome, that these microbiome alterations may increase the risk for infections with *Clostridioides difficile.* An oralization of the stool microbiome in SC-CIP with species that are normally abundant in the mouth and the upper gastrointestinal tract became evident. *Rothia dentocariosa*, a bacterium normally found in the mouth of healthy humans with a potential pathogenic potential [62], and *Streptococcus parasanguinis*, a species associated with a healthy oral microflora [63], were elevated in the stool of SC-CIP. The partially antibiotic-resistant pathogen *Enterococcus faecium* [64] was more abundant in SC-CIP, which was also previously described in long-stay ICU patients [44]. In SC-CIP, the probiotic strain *Streptococcus thermophiles* was elevated, and in healthy controls, the butyrate producing strain *Anaerostipes hadrus* was elevated. Butyrate plays a crucial role in the gut as one of the most important metabolites, being an energy source of the intestinal epithelium and inhibiting inflammation in a healthy gut. In a mouse model, *Anaerostipes hadrus* showed the ability to harm the inflamed intestinal epithelium, suggesting that bacterial strains can play different roles depending on the setting [65].

A proportion of critically ill patients at an ICU need long-term treatment, sometimes over weeks, with invasive ventilation, vasoactive drugs and invasive procedures. Further on, they receive multiple medications including antibiotics, antiviral and antifungal therapies and enteric or parenteral nutrition. That all these interventions together with the underlying critical illness alter the microbiome on different body sites has been shown recently, as well as that these changes may trigger hospital-acquired infections, sepsis, multi-organ failure, energy homeostasis disturbance, muscle wasting and cachexia [10,12,13,44,66,67,68]. One reason for this may be the slower bowel transit time in critically ill patients caused by electrolyte and glucose disturbances as well as by therapeutics like sedatives and opiates [66]. Therefore, therapeutic efforts with approaches such as probiotics, symbiotics or fecal microbiota transplants have been investigated [68,69,70,71,72,73,74]. Our cohort of critically ill patients who did not develop SC-CIP had all been discharged from ICU a year before study inclusion on average, but still, taxonomic differences remained on all levels compared to healthy controls. The reduction of *Faecalibacterium prausnitzii*, a species thought to be beneficial for survival and function of enterocytes due to the production of short-chain fatty acids [75] and known to show reduced abundance in many intestinal disorders [76], as well as elevation of *Erysipelatoclostridium ramosum*, a species involved in the development of metabolic syndrome [77], were observed. With the knowledge that both SC-CIP and CIP controls independent of the presence of a liver disease show dysbiosis compared to healthy controls, the question remains if certain microbial differences could trigger the development of SC-CIP. Indeed, we could show that the microbiome of these two cohorts differs widely. A shift of highly abundant classes like *Bacilli* and *Bacteroidia* could be shown as well as changes going down to the species level, with reduction of an unnamed *Neisseria* species and *Prevotella melaninogenica* in SC-CIP. Dysbiosis of the intestinal microbiome is a known feature of alcohol-induced cirrhosis. *Bifidobacteria, Streptococci, Veillonella* and *Enterobacteria* were found to be elevated in cirrhosis, whereas *Faecalibacterium* showed a reduction [58,76,78]. In our cohort, we could observe reduced diversity, and on a genus level, also increased abundance of *Veillonella* compared to healthy controls. This species could cause joint infections, endocarditis and was associated with hepatic encephalopathy [66,67,68], but can also be an effect of PPI intake of some cirrhotic patients [79].

Alterations of the gut microbiome lead to increased inflammation, a leaky gut and endotoxemia, not only in gastrointestinal pathologies but also in liver disease [80]. In our study, we could demonstrate this association and potential pathophysiological link for the first time in SC-CIP. Compared to healthy controls, SC-CIP patients had higher biomarkers of gut permeability (DAO), systemic inflammation (CRP) and bacterial translocation (sCD14), although none of the patients had clinically apparent signs of infection when the samples were taken. DAO can be found in several human tissues but is particularly present in the intestine and correlates with the integrity of the intestinal mucosa. Elevated DAO levels are a serum biomarker for increased intestinal permeability [81]. sCD14 is an LPS binding glycoprotein which can be found on the surface of macrophages and monocytes and is produced during inflammation and sepsis. Elevated serum levels are considered to be a surrogate biomarker for endotoxemia [82]. Our study suggests that this may be a consequence of the intensive care treatment rather than an effect of the liver disease alone, since increased gut permeability could also be shown in CIP controls. Enterocyte damage plays a role in the outcome of critically ill patients, as suggested by increased plasma intestinal fatty acid-binding protein and citrulline concentrations in plasma [83]. The positive correlation of inflammation and gut permeability markers with important parameters of liver dysfunction, as observed in our current study, suggests a connection between gut inflammation and the severity of the liver disease. This was also shown previously by our study group [84].

When searching for factors that may connect dysbiosis and intestinal wall integrity in a cholestatic disease, bile acids need to be considered. Bile acids consist of steroid molecules and can be divided into primary bile acids, which are produced in the liver, and into secondary bile acids, which are the result of primary bile acids being metabolized by the gut microbiome [85]. This bile acid homeostasis is controlled by the intestinal microbiome [86]. Thus, changes in the microbiome by inflammation, diet or antibiotic intake may also lead to changes in the bile acid profile [87]. As expected, in a cholestatic liver disease, an increase in total serum bile acids was found in SC-CIP. This has previously also been published for other cholestatic liver diseases, such as primary biliary cholangitis, primary sclerosing cholangitis and NAFLD [88,89]. Furthermore, bile acid profiles were altered in SC-CIP and cirrhosis compared to healthy controls. Other than in primary and secondary, bile acids can also be divided into conjugated and unconjugated bile acids. This conjugation in adults aims to improve hydrophily and protects against toxic effects of the bile acids. In the intestine, primary conjugated bile acids are deconjugated by bacterial bile salt hydrolase [90,91]. As in primary biliary cholangitis and primary sclerosing cholangitis, conjugated bile acids were elevated in SC-CIP and cirrhosis serum [89]. An increase in taurine-conjugated bile acids in gall bladder aspirates of mice was associated with the expansion of pathobionts and the development of colitis [92], and may therefore be part of the cause and/or consequence of the increase of potential harmful bacterial species. Germ-free or antibiotic-treated rodents also showed an elevation of taurine-conjugated primary BA species in plasma and in the liver; therefore, the observed elevation of conjugated bile acids in the SC-CIP population may be linked to the loss in microbiome diversity and antibiotic use in critically ill patients [93].

Our study has several important limitations. The cross-sectional study design does not allow to establish causal relationships. SC-CIP is a rare disease; therefore, the sample size is rather small, although three centers contributed to the study. Also, recruiting patients as CIP controls was challenging. Collection of stool samples at only one time point from each proband makes the analysis more vulnerable for daily fluctuations of the microbiome, and the cross-sectional design does not allow conclusions on longitudinal effects. Furthermore, 16S sequencing, although the current standard method for microbiome analysis, provides less microbial resolution compared to whole metagenome sequencing techniques, which can lead to considerable limitations in species level identification. Therefore, only those sequences which unambiguously and exactly matched a reference genome were analyzed on this level.

## 5. Conclusions

In conclusion, our study implies that SC-CIP is associated with dysbiosis of the gut microbiome, showing severe structural and functional changes in microbiome composition. Oralization of the gut microbiome and an increase of potential harmful bacteria were observed. Furthermore, increased gut permeability, bacterial translocation, intestinal and systemic inflammation, as well as an alteration of the serum bile acid profile, were observed. Our study also suggests that these alterations are not only associated with the liver disease itself, since long-term ICU treatment with invasive ventilation and catecholamine treatment also leaves an important long-lasting mark on the microbiome and gut permeability. Bile acids may be a potential diagnostic and therapeutic target in SC-CIP. Further studies are needed to evaluate the microbiome changes longitudinally and to identify potential points of action of microbiome-modulating therapies that could improve the outcome of this devastating disease.

## Figures and Tables

**Table 1:** Baseline characteristics for Secondary Sclerosing Cholangitis in Critically Ill Patients (SC-CIP), patients after critical illness without liver disease (CIP controls), cirrhosis and healthy controls including age, sex, body mass index, most frequently taken drugs during sample acquisition and relevant laboratory markers

Characteristics	SC-CIP (*n* = 18)	CIP Controls (*n* = 11)	Cirrhosis (*n* = 21)	Healthy (*n* = 21)	*p*-Value
Age (years) ^†^	59 ± 12.7 years	54 ± 15 years	58 ± 9 years	58 ± 6.9 years	0.827
Sex (male/female)	13/5	8/3	16/5	9/12	0.204
Body Mass Index ^†^	27.2 ± 5.3	25.5 ± 3	27.3 ± 5	25.3 ± 3.1	0.350
Protone pump inhibitors (yes/no)	6/12	2/9	13/8	2/19	0.002
Antihypertensives (yes/no)	8/10	1/10	4/17	10/11	0.114
NSAIDS (yes/no) ^‡^	0/18	3/8	5/16	2/19	0.088
Platelet function inhibitors (yes/no)	4/14	5/6	0/21	1/20	0.002
Statins (yes/no)	3/15	2/9	1/20	1/20	0.388
Thyroid medication (yes/no)	4/14	5/6	1/20	4/17	0.054
Beta blockers (yes/no)	6/12	2/9	14/7	5/16	0.004
Alkaline phosphatase (U/l) ^†^	287.4 ± 262.2	89.8 ± 20.9	111.5 ± 47.2	61.8 ± 14.8	<0.001
Gamma-glutamyl-transferase (U/l) ^†^	518.1 ± 371.2	34.6 ± 15.3	213.6 ± 182.1	21.2 ± 10.5	<0.001
Bilirubin (mg/dl) ^†^	2.0 ± 3.2	0.3 ± 0.2	1.2 ± 0.6	0.4 ± 0.8	0.02
Creatinine (mg/dl) ^†^	1.0 ± 0.4	0.6 ± 0.2	0.9 ± 0.3	0.9 ± 0.2	0.2
CRP (mg/l) ^§,†^	12.7 ± 13.6	6.4 ± 7.2	3.7 ± 3,7	2.1 ± 2.2	<0.001
INR ^¶,†^	1.2 ± 0.4	1.0 ± 0.09	1.2 ± 0.3	1.0 ± 0.04	0.001

^†^ Data are expressed as the mean ± standard deviation (SD). ^‡^ non-steroidal anti-inflammatory drugs; ^§^ c-reactive protein; ^¶^ international normalized ratio.

**Table 2:** ICU treatment and disease characteristics for Secondary Sclerosing Cholangitis in Critically Ill Patients (SC-CIP) and patients after critical illness without liver disease (CIP controls).

Characteristics	SC-CIP (*n* = 18)	CIP-Controls (*n* = 11)	*p*-Value
Cirrhosis/Fibrosis/None	7/4/7	0/0/11	0.004
ICU treatment (days) ^†,‡^	42 ± 20	19 ± 12	0.001
Mean ventilation time (days) ^†^	28 ± 18	14 ± 9	0.036
Catecholamine treatment (days) ^†^	12 ± 8	n.a.	n.a.
Interval ICU admission—SC-CIP diagnosis (days) ^†^	311 ± 441	n.a.	n.a.
Hemodialysis (yes/no)	5/13	1/10	0.228
Treatment with UDCA (yes/no) ^§^	12/6	0/11	<0.001
Death (yes/no)	0/18	0/11	n.a.
Liver transplantation (yes/no)	1/17	0/11	0.426

^†^ Data are expressed as the mean ± SD. ^‡^ intensive care treatment; ^§^ ursodeoxycholic acid; n.a. = not available.

**Table 3:** The table shows results of the two used multivariate models (RDA+) to outline microbiome-influencing factors. Group assignment, statin intake, platelet function inhibitor intake, BMI, calprotectin and cirrhosis remained possible explanatory variables for microbiome perturbations, with only statin intake being significant in both models. Significant confounders are marked in bold. In column one of each model, the variance is shown, in column two, results of the F-test are shown and in column 3, the *p*-value is indicated.

	Model 1			Model 2		
	Variance	F	*p*	Variance	F	*p*
**Group**	82.62	1.90	**0.001**			
Proton pump inhibitors	15.39	1.06	0.220	16.76	1.06	0.194
**Statins**	18.85	1.30	**0.007**	24.38	1.54	**0.001**
Beta Blockers	15.13	1.05	0.250	16.61	1.05	0.245
**Platelet function inhibitors**	15.03	1.04	0.304	24.09	1.52	**0.001**
**Body mass index**	14.58	1.01	0.496	24.21	1.53	**0.001**
CRP ^†^	13.51	0.93	0.751	15.11	0.95	0.673
INR ^‡^	15.29	1.06	0.225	16.43	1.04	0.314
**Calprotectin**	16.10	1.11	0.102	19.54	1.23	**0.022**
DAO ^§^	14.01	0.97	0.600	15.85	1.00	0.464
LBP ^¶^	14.03	0.97	0.623	15.05	0.95	0.678
Zonulin	13.00	0.90	0.846	15.34	0.97	0.620
**Cirrhosis**				22.46	1.42	**0.003**
sCD14 ^¶¶^				13.73	0.87	0.924

^†^ c-reactive protein; ^‡^ international normalized ratio; ^§^ diaminoxidase; ^¶^ lipopolysaccharide-binding protein; ^¶¶^ soluble cluster of differentiation 14.

**Table 4:** Shown are possible explanatory variables for differences in microbiome composition of SC-CIP and CIP controls, but all three variables dropped out in the multivariate analysis with RDA+ and only group assignment remained. Significant confounders are marked in bold. In column one, the variance is shown, in column two, results of the F-test are shown and in column 3, the *p*-value is indicated

	Variance	F	*p*
**Group**	57.99	1.40	**0.001**
Body mass index	29.89	0.72	0.844
Bilirubin	58.02	1.40	0.094
sCD14 ^†^	32.57	0.79	0.947

^†^ soluble cluster of differentiation 14.

**Table 5:** Taxonomic differences found with analysis of composition of microbiomes between SC-CIP and healthy groups on all taxonomic levels.

SC-CIP vs. Healthy		
Phylum	SC-CIP	Healthy
Actinobacteria	3.19	0.62
Bacteriodetes	28.94	43.27
Patescibacteria	0.039	0.0046
Tenericutes	0	0.17
Class		
Alphaproteobacteria	0.033	0.57
Bacilli	8.99	0.4
Order		
Betaproteobacteriales	0.92	1.38
Izimaplasmatales	0	0.16
Lactobacillales	8.95	0.4
Micrococcales	0.096	0.015
Rhodospirillales	0.033	0.57
Family		
Carnobacteriaceae	0.023	0.0043
Christensenellaceae	0.5	3.01
Enterococcaceae	3.17	0.022
Micrococcaceae	0.097	0.015
Streptococcaceae	4.34	0.35
Genus		
Actinomyces	0.17	0.019
Anaerostipes	0.17	1.43
Atopobium	0.042	0.0019
Bifidobacterium	0.021	0.0022
Coprococcus_2	0.033	0.67
Enterococcus	3.3	0.023
Lachnospira	0.13	0.27
Lactobacillus	1.43	0.025
Lactococcus	0.14	0.013
Rothia	0.1	0.016
Ruminiclostridium	0.047	0.87
Ruminococcaceae_UCG005	0.14	0.78
Ruminococcaceae_UCG014	0.87	2.9
Sellimonas	0.29	0.024
Solobacterium	0.023	0.0024
Streptococcus	4.46	0.37
Sutterella	0.29	1.05
OTU ^†^		
Streptococcus thermophilus	0.69	0.033
Species		
Streptococcus parasanguinis	0.33	0.042
Rothia dentocariosa	0.029	0
Sellimonas intestinalis	0.074	0.0024
Enterococcus faecium	0.37	0
Anaerostipes hadrus	0.072	0.87

Data are expressed as arithmetic mean. ^†^ operational taxonomic unit.

**Table 6:** Taxonomic differences found with analysis of composition of microbiomes between CIP controls and the healthy group on all taxonomic levels.

CIP Controls vs. Healthy		
Phylum	CIP controls	Healthy
Firmicutes	49.45	53.21
Fusobacteria	0.03	0.0019
Proteobacteria	1.48	2.67
Synergistetes	0	0.00087
Class		
Bacteriodia	47	43.43
Clostridia	41.04	47.83
Gammaproteobacteria	1.63	1.8
Order		
Betaproteobacteriales	0.87	1.38
Micrococcales	0.041	0.015
Family		
Micrococcaceae	0.041	0.015
Neisseriaceae	0.032	0.00083
Genus		
Agathobacter	0.51	1.94
Coprococcus_2	0.12	0.57
Eisenbergiella	0.32	0.015
Faecalibacterium	1.47	5.9
Neisseria	0.056	0.0009
Sellimonas	0.44	0.024
Species		
Erysipelatoclostridium ramosum	0.65	0.012
Faecalibacterium prausnitzii	1.28	5.91

Data are expressed as arithmetic mean.

**Table 7:** Taxonomic differences found with analysis of composition of microbiomes between cirrhosis and healthy groups on all taxonomic levels.

Cirrhosis vs. Healthy		
Phylum	Cirrhosis	Healthy
Synergistetes	0.0013	0.00017
Class		
Clostridia	36	47.83
Erysipelotrichia	1.3	2.61
Oxyphotobacteria	0.0032	0.00064
Synergistia	0.0013	0.00088
Order		
Bacteroidales	45.78	43.41
Betaproteobacteriales	1.24	1.38
Clostridiales	36.02	47.84
Corynebacteriales	0.00031	0.00029
Erysipelotrichales	1.3	2.61
Family		
Christensenellaceae	0.72	3.01
Clostridiaceae_1	0.13	0.9
Peptostreptococcaceae	0.82	2.83
Genus		
Coprococcus_2	0.025	0.67
Romboutsia	0.22	1.13
Veillonella	0.4	0.033
OTU ^†^		
Uncultured Bacterium	0.09	0.28
Uncultured Bacterium	0.00075	0.2
Species		
Coprococcus_2_NA	0.023	0.5
Romboutsia_NA	0.18	1.01

Data are expressed as arithmetic mean. ^†^ operational taxonomic unit.

**Table 8:** Taxonomic differences found with analysis of composition of microbiomes between the SC-CIP group and CIP controls on all taxonomic levels.

SC-CIP vs. CIP Controls		
Phylum	SC-CIP	CIP controls
Firmicutes	64.1	49.45
Patescibacteria	0.039	0.00053
Tenericutes	0	0.047
Class		
Bacilli	8.99	3.45
Bacteroidia	28.91	47
Mollicutes	0	0.047
Saccharimonadia	0.039	0.00053
Family		
Neisseriaceae	0	0.032
Genus		
Anaerotruncus	0.01	0.42
Bifidobacterium	0.021	0.0052
Coprobacillus	0.044	0.26
Fusicatenibacter	1.65	0.51
Intestinimonas	0.019	0.14
Neisseria	0	0.036
Oscillibacter	0.13	0.77
Prevotella_7	0	0.038
Solobacterium	0.023	0.0023
OTU ^†^		
Uncultured Bacterium	0.042	0.71
Species		
Neisseria_NA	0	0.032
Prevotella_7_melaninogenica	0	0.03

Data are expressed as arithmetic mean. ^†^ operational taxonomic unit.

**Table 9:** Taxonomic differences found with analysis of composition of microbiomes between SC-CIP and cirrhosis groups on all taxonomic levels.

SC-CIP vs. Cirrhosis		
Phylum	SC-CIP	Cirrhosis
Actinobacteria	3.19	1.21
Bacteroidetes	28.94	45.86
Firmicutes	64.1	46.83
Patescibacteria	0.039	0.0002
Tenericutes	0	0.018
Class		
Alphaproteobacteria	0.033	1.69
Mollicutes	0	0.018
Order		
Rhodospirillales	0.033	1.69
Family		
Atopobiaceae	0.57	0.0074
Bifidobacteriaceae	0.018	0
Eggerthellaceae	0.59	0.069
Erysipelotrichaceae	3.51	1.34
Lachnospiraceae	25.49	21.57
Peptostreptococcaceae	5.95	0.82
Genus		
Actinomyces	0.17	0.048
Anaerostipes	0.17	2.44
Bifidobacterium	0.021	0
Dorea	1.52	0.43
Lachnospira	0.13	0.29
Solobacterium	0.023	0.0028
Species		
Anaerostipes hadrus	0.072	2.04

Data are expressed as arithmetic mean.

**Table 10:** Taxonomic differences found with analysis of composition of microbiomes between CIP controls and the cirrhosis group on all taxonomic levels.

CIP Controls vs. Cirrhosis		
Phylum	CIP controls	Cirrhosis
Firmicutes	49.45	46.83
Proteobacteria	1.48	6.06
Synergistetes	0	0.0013
Class		
Alphaproteobacteria	0.3	1.69
Clostridia	41.04	36
Erysipelotrichia	2.55	1.3
Gammaproteobacteria	1	3.99
Synergistia	0	0.0013
Order		
Bacteriodales	47	45.78
Clostridiales	41.04	36.02
Corynebacteriales	0	0.00031
Erysipelotrichiales	2.55	1.3
Propionibacteriales	0.00068	0.0074
Rhodospirillales	0.3	1.69
Synergistales	0	0.0013
Family		
Eggerthellaceae	0.51	0.069
Neisseriaceae	0.032	0.00073
OTU ^†^		
Uncultured Bacterium	0.0026	0.25
Uncultured Bacterium	0.11	0.012
Uncultured Bacterium	0.61	0

Data are expressed as arithmetic mean. ^†^ operational taxonomic unit.

**Table 11:** Heatmap showing correlations between markers of gut permeability, bacterial translocation, intestinal and systemic inflammation and markers for liver function, kidney function, age and BMI. Spearman’s correlation coefficient is shown. Significant results (*p* < 0.05) are indicated by *. The greener a field gets, the better the positive correlation, the more red a field gets, the more pronounced the negative correlation is.

	Zonulin	DAO ^†^	sCD14 ^‡^	LBP ^§^	CRP ^¶^	Calprotectin
Creatinine	−0.042	−0.074	−0.052	0.147	0.047	0.019
Bilirubin	0.037	0.226	0.432 *	0.27 *	0.554 *	0.029
INR ^¶¶^	0.107	0.286 *	0.339 *	−0.013	−0.08	0.092
Liver stiffness	−0.207	0.629 *	0.268	0.512 *	0.52 *	0.457
Age	−0.023	0.026	0.015	0.14	0.229	0.05
Body mass index	−0.03	0.32 *	0.226	0.073	0.235 *	−0.081

^†^ diaminoxidase; ^‡^ soluble cluster of differentiation 14; ^§^ lipopolysaccharide-binding protein; ^¶^ c-reactive protein; ^¶¶^ international normalized ratio.

**Table 12:** Heatmap showing correlations between bile acid profiles and markers of gut permeability, bacterial translocation, intestinal and systemic inflammation and liver function parameters. Spearman’s correlation coefficient is shown. Significant results (*p* < 0.05) are indicated by *. The greener a field gets, the better the positive correlation, the more red a field gets, the more pronounced the negative correlation is.

	Total	Primary/	Unconjugated/	Primary	Secondary	Total Conjugated	Total Unconjugated
	Bile Acids	Secondary Bile Acids	Conjugated Bile Acids	Bile Acids	Bile Acids	Bile Acids	Bile Acids
sCD14 ^†^	0.529 *	0.108	0.562 *	0.109	0.196	0.517 *	0.093
Zonulin	−0.011	−0.149	−0.041	−0.059	0.031	0.14	0.111
DAO ^‡^	0.367 *	0.287 *	0.217	0.309 *	0.123	0.331 *	0.283 *
LBP ^§^	0.1	−0.08	0.117	−0.107	0.032	0.106	−0.125
CRP ^¶^	0.143	−0.008	0.131	−0.049	−0.13	0.133	−0.033
Calprotectin	0.028	−0.076	0.293 *	−0.016	0.035	0.011	−0.034
Creatinine	−0.062	−0.221	−0.051	−0.204	0.095	−0.066	−0.171
Bilirubin	0.268 *	0.088	0.391 *	−0.014	−0.067	0.294 *	−0.049
INR ^¶¶^	0.353 *	0.109	0.308 *	0.102	0.34 *	0.303 *	0.098
Liver stiffness	0.194	0.193	0.64 *	0.293	−0.33	0.356	−0.305

^†^ soluble cluster of differentiation 14; ^‡^ diaminoxidase; ^§^ lipopolysaccharide-binding protein; ^¶^ c-reactive protein; ^¶¶^ international normalized ratio.

## Abbreviations

ANCOM	analysis of composition of microbiomes
ANOVA	analysis of variance
BMI	body mass index
CIP	critically ill patients
CRP	c-reactive protein
DAO	diaminoxidase
DNA	deoxyribonucleic acid
ELISA	Enzyme-Linked Immunosorbent Assay
ERCP	endoscopic retrograde cholangiopancreatography
F/B ratio	Firmicutes/Bacteriodetes ratio
FDR	false discovery rate
HCV	hepatitis C virus
IBD	inflammatory bowel disease
ICU	intensive care unit
INR	international normalized ratio
kPa	kilopascal
LBP	lipopolysaccharide-binding protein
MELD	model of end-stage liver disease
MRCP	magnetic resonance cholangiopancreatography
NAFLD	non-alcoholic fatty liver disease
NSAIDS	non-steroidal anti-inflammatory drugs
NMMDS	non-metric multidimensional scaling
OTU	operational taxonomic unit
PPI	proton pump inhibitor
PCR	polymerase chain reaction
PSC	primary sclerosing cholangitis
RDA	redundancy analysis
SC-CIP	secondary sclerosing cholangitis in critically ill patients
sCD14	soluble cluster of differentiation 14
UDCA	ursodeoxycholic acid
VIF	variance inflation factor
